# Refractory Auto-Immune Thrombotic Thrombocytopenic Pupura Successfully Treated With Caplacizumab

**DOI:** 10.3389/fmed.2020.549931

**Published:** 2020-10-22

**Authors:** Chloé Mellaza, Nicolas Henry, Pierre-Marie Fayolle, Satar Mortaza, Jean-François Subra, Agnès Veyradier, Paul Coppo, Jean-François Augusto

**Affiliations:** ^1^Service de Néphrologie-Dialyse-Transplantation, Centre Hospitalier Universitaire Angers, Angers, France; ^2^Département de Réanimation Médicale et Médecine Hyperbare, Centre Hospitalier Universitaire Angers, Angers, France; ^3^Université d'Angers, Angers, France; ^4^Service d'Hématologie Biologique, Hôpital Lariboisière and EA3518, Hôpital Saint Louis, Groupe Hospitalier Saint Louis-Lariboisière, Assistance Publique-Hôpitaux de Paris, Paris, France; ^5^Center de Référence des Microangiopathies Thrombotiques, Paris, France; ^6^Service d'Hématologie Hôpital Saint-Antoine AP-HP, Paris, France

**Keywords:** thrombotic thrombocitopenic purpura, caplacizumab, refractor, case report, platelet

## Abstract

Thrombotic thrombocytopenic purpura (TTP) is a rare thrombotic microangiopathy characterized by mechanical hemolytic anemia, profound thrombocytopenia, and neurological manifestations. Acquired auto-immune TTP, the most prevalent cause of TTP, is induced by the presence of inhibitory anti-ADAMTS13 auto-antibodies. Modern treatment of acquired TTP relies on plasma exchange, rituximab, and steroids. Caplacizumab (Cablivi®), a humanized single-variable domain immunoglobulin that targets the A1 domain of the ultra-large von Willebrand factor, inhibits the interaction between ultra-large vWFand platelets. In two clinical trials, caplacizumab, in addition to conventional treatment, shortened the delay to platelet count normalization in comparison to conventional treatment plus placebo, without increasing significantly hemorrhagic complications. Moreover, caplacizumab was associated with reduced occurrence of a secondary endpoint associating death, TTP recurrence, and major thromboembolic events. Here, we report the off-label use of caplacizumab in a 68-year-old patient with confirmed acquired TTP, severe thrombocytopenia, and generalized tonic–clonic seizures requiring mechanical ventilation and admission in the intensive care unit. Conventional treatment was rapidly started. Despite the intensification of plasma exchange treatment with twice-daily sessions, steroid continuation, and a second rituximab infusion on day 6, thrombotic microangiopathy worsened with thrombocytopenia at 21 g/L on day 8 from admission. We also considered using caplacizumab, which we could obtain and start on day 12 from admission, as it was available under a temporary authorization use in France. As soon as 12 h after caplacizumab initiation, we observed a significant increase of platelet count and improvement of other hemolytic parameters. We observed resolution of encephalopathy and complete recovery of motor paralysis, allowing us to stop mechanical ventilation on day 14. Caplacizumab was maintained for 128 days until day 139 from initial admission. The patient is going well 10 months after initial admission, without any neurological sequelae, and TTP did not relapse. To the best of our knowledge, this is the first reported use of caplacizumab in such a condition. This case report suggests that caplacizumab use may help to reduce the rate of refractory TTP episodes.

## Background

Thrombotic thrombocytopenic purpura (TTP) is a rare thrombotic microangiopathy characterized by mechanical hemolytic anemia, profound thrombocytopenia, and neurological manifestations (1). Acquired auto-immune TTP, the most prevalent cause of TTP, is induced by the presence of inhibitory anti-ADAMTS13 auto-antibodies. At the pathophysiological level, auto-antibodies induce severe ADAMTS13 deficiency (typically below 10%) and the inability to cleave von Willebrand factor (vWF), the ADAMTS13 substrate, leading to the expression of prothrombotic ultra-large vWF in the microcirculation. Interactions between ultra-large vWF and platelets induce microthrombi formation, which is responsible for tissue injury and organ dysfunction (1).

Modern treatment of acquired TTP relies on plasma exchange (PE), rituximab (RTX), and steroids (2). PE allows to remove autoantibodies and to replenish ADAMTS13 concentration using plasma from healthy donors, whereas RTX targets B cells, including autoreactive B cells that produce pathogenic autoantibodies. Using this regimen, up to 85% of patients achieve disease remission by 5–15 days, the latter being classically defined as organ dysfunction recovery and sustained platelet count normalization. In a minority of patients, resistance to first-line treatment is observed, leading to consider the use of intensive PE (twice daily), cytotoxic agents (i.e., cyclophosphamide), and/or splenectomy (3). It is important to underline that despite significant therapeutic signs of progress, acquired TTP remains a life-threatening disease with a mortality rate ranging from 5 to 15% occurring predominantly within the 2 weeks after hospital admission (4).

Caplacizumab (Cablivi®), a humanized single-variable domain immunoglobulin that targets the A1 domain of the ultra-large vWF, inhibits the interaction between ultra-large vWF and platelets (5). In two clinical trials, caplacizumab, in addition to conventional treatment, allowed to shorten the delay to platelet count normalization in comparison with conventional treatment plus placebo, without increasing significantly hemorrhagic complications (6, 7). Moreover, caplacizumab was associated with reduced occurrence of a secondary endpoint associating death, TTP recurrence, and major thromboembolic events (7). After these trials, caplacizumab was approved for TPP treatment and conventional treatment in the US and Europe.

Here, we report the off-label use of caplacizumab in a patient with acquired TTP resistant to conventional therapy. To the best of our knowledge, this is the first reported use of caplacizumab in such a condition.

## Case Presentation

A 68-year-old woman was admitted to the emergency department of a local hospital after a 15-min transient neurological attack. She had a history of essential hypertension treated with lercanidipine and Sjögren's syndrome, which was recently diagnosed. On examination, arterial pressure was 148/85 mmHg; she had purpuric lesions of the upper limbs, petechia of the mucosa, and diffuse ecchymoses. The neurological examination was first normal. Biological examination revealed thrombotic microangiopathy (TMA), with mechanical microangiopathic hemolytic anemia (hemoglobin 8.9 g/dL, haptoglobin below 0.2 g/L, free bilirubin at 57 μmol/L, lactate dehydrogenase 1,245 UI/L, schistocytes between 5 and 10%, and thrombocytopenia at 10 g/L). Serum creatinine was 94 μmol/L, without significant proteinuria. The diagnosis of TTP was rapidly considered, and she was transferred to the proximity university hospital for therapeutic management.

Confirming acquired TTP, ADAMTS13 activity was below 5%, and anti-ADAMTS13 antibodies (titer > 100 UI/ml) were detected. Positive antinuclear antibodies (1/200) were also detected with anti-Sm-RNP antibodies. The search for antiphospholipid antibody syndrome was negative.

After the first PE session on day 1, the patient presented generalized tonic–clonic seizures requiring mechanical ventilation and admission to the intensive care unit. Steroids (1-mg/kg/day prednisone) and the first infusion of 375-mg/m^2^ RTX were administered. Her clinical and biological condition improved on day 3, allowing to stop sedative drugs and weaning from mechanical ventilation. However, despite PE treatment, steroid continuation, and a second RTX infusion on day 6, TMA worsened with thrombocytopenia at 21 g/L on day 8 from admission. At that time, we decided to intensify PE treatment to twice-daily sessions. However, her clinical and neurological condition worsened. Indeed, TMA was not controlled as attested by the platelet count remaining below 10 g/L. On day 9, she developed encephalopathy with repetitive partial seizures necessitating profound sedation and mechanical ventilation. At clinical examination, persistent motor paralysis of the upper left limb was observed. The CT scan was normal, excluding brain hemorrhage, and the MRI showed two micro-infarctions. A third RTX infusion was done on day 10. On day 11, platelet count was 7 g/L, and encephalopathy with motor paralysis was persistent. Given that complement activation has been observed in TMA, including in TTP, and based on some literature case reports (8), we decided to administer eculizumab to our patient as a rescue therapy on day 11. Despite complement blockade, we did not observe any improvement of TMA activity and neurological involvement.

Concomitantly, we also considered using caplacizumab, which we could obtain and start on day 12 from admission, as it was just available under a temporary authorization use in France. The first injection of caplacizumab was done intravenously at 10-mg dose, followed by 10 mg twice-daily subcutaneous administration after each PE session, as recommended by the manufacturer. As soon as 12 h after caplacizumab initiation, we observed a significant increase of platelet count and improvement of other hemolytic parameters. We observed a resolution of encephalopathy and complete recovery of motor paralysis, allowing to stop mechanical ventilation on day 14. A drop of platelet count to 66 g/L without other biological signs of TMA occurred on day 18, which we attributed to a hypersensibility reaction to plasma infusion. Complete resolution of biological TMA signs was observed on day 24 from admission.

**Figure 1:**
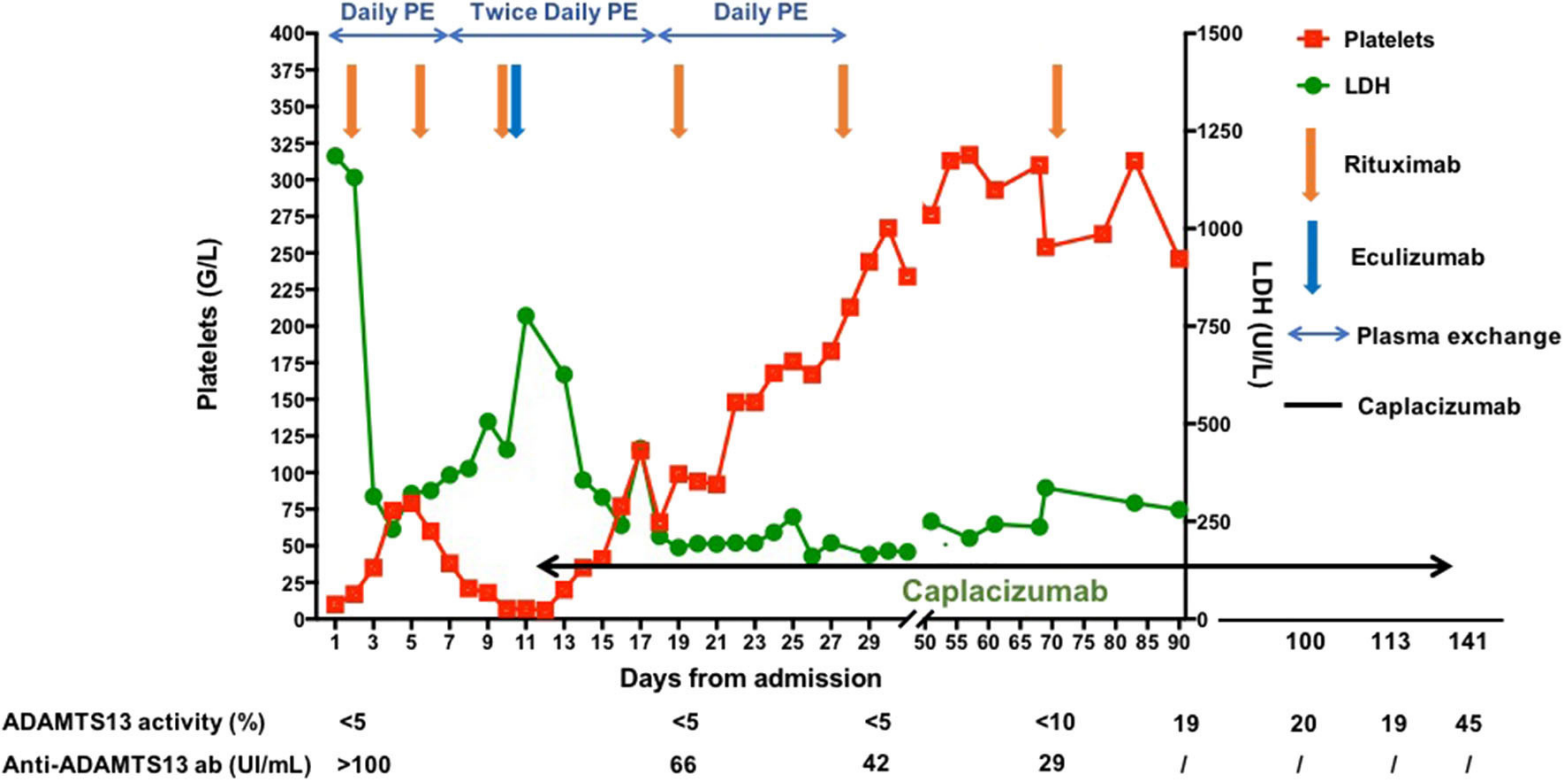
Treatment management and evolution of the platelet count and the lactate dehydrogenase level from diagnosis and day 90. The patient gave her written consent for publication of the case report.

PE sessions were continued twice daily until day 20 from admission and could be stopped on day 31 (a total of 41 PE sessions performed). Rituximab infusions were administered on day 19 (CD19 count 17 cells/μl, fourth infusion), day 28 (CD19 count 2 cells/μl, fifth infusion), and day 71 (CD19 count 1 cell/μl, sixth infusion) to achieve complete B cell depletion. In parallel, we closely monitored ADAMTS13 activity and decided to continue caplacizumab until ADAMTS13 activity returned above 20%. Caplacizumab was maintained for 128 days until day 139 from initial admission. Apart from a benign palpebral hematoma, no other adverse event occurred under caplacizumab treatment. The treatment management and the evolution of TMA parameters are summarized in Figure 1. The patient was going well 20 months after initial admission, without any neurological sequelae and did not develop TTP relapse.

## Discussion

Caplacizumab has been tested in two clinical trials as an add-on therapy to conventional TTP treatment associating PE, steroids, and RTX (6, 7). Both trials were associated with faster thrombocytopenia correction in the caplacizumab groups. Interestingly, in the phase III trial HERCULES, no patient in the caplacizumab group experienced refractory TTP defined by the absence of platelet count doubling after 4 days of treatment, whereas three patients did in the conventional treatment group. At the difference of clinical trials where it was tested, caplacizumab was used as rescue therapy for a severe resistant form of TTP in our patient. Interestingly, platelet count increased very early after caplacizumab initiation, suggesting an immediate action of the molecule at the microcirculation level. We observed concomitantly a fast correction of the hemolytic parameters and clinical improvement of the patient. Thus, these observations clearly suggest that caplacizumab can improve microcirculation and prevent tissue damage.

Consequently, as illustrated in our case, the ability of caplacizumab to control very severe TTP forms strengthens the view that its use, along with first-line treatment, should reduce significantly life-threatening forms of the disease and the need for more aggressive salvage therapies (twice daily PE, cyclophosphamide, splenectomy). Finally, in our patient, if caplacizumab were not available to use, we would have considered cyclophosphamide administration or splenectomy. Both have been reported in the literature as salvage therapies that should be considered alone or sequentially in patients with severe and resistant TTP forms (9, 10). However, the use of these treatments relies mainly on small retrospective studies, and their safety remains controversial, especially in regard to bleeding risk after splenectomy and infectious complications (9, 10).

## Ethics Statement

The patient gave her written, informed consent for publication of the case report.

## Author Contributions

CM and NH: took care of the patient and wrote the first version of manuscript. P-MF and SM: took care of the patient in the ICU. J-FS: revised the manuscript. AV: did the ADAMTS13 measurement. PC: revised the manuscript and helped in treatment decision. J-FA: wrote and revised the manuscript, took care of the patient, and derived treatment decisions. All authors contributed to the article and approved the submitted version.

## Conflict of Interest

The authors declare that the research was conducted in the absence of any commercial or financial relationships that could be construed as a potential conflict of interest.
